# Associations of the Severity of Obstructive Sleep Apnea With Age-Related Comorbidities: A Population-Based Study

**DOI:** 10.3389/fneur.2022.802554

**Published:** 2022-05-10

**Authors:** Mayra dos Santos Silva, Dalva Poyares, Luciana Oliveira Silva, Ksdy M. Souza, Monica L. Andersen, Maurice M. Ohayon, Sergio Tufik, Ronaldo D. Piovezan

**Affiliations:** ^1^Department of Psychobiology, Universidade Federal de São Paulo, São Paulo, Brazil; ^2^Psych/Public Mental Health and Population Sciences, Stanford University, Stanford, CA, United States

**Keywords:** sleep apnea, aging, older adults, hypertension, body mas index

## Abstract

Aging is accompanied by changes in the quantity and quality of sleep. Obstructive sleep apnea (OSA) is also more prevalent in the older population. Although severe OSA has been linked to a higher risk of cardiovascular disease regardless of adult age, clinical consequences of mild-to-moderate OSA in the older adults are still uncertain.

**Objectives:**

To investigate the relationships between severity and metabolic, cognitive, and functional characteristics in community-dwelling older adults from a representative sample of the city of São Paulo.

**Methods:**

In total, 199 participants of the first follow-up of the São Paulo Epidemiologic Sleep Study (EPISONO, São Paulo, Brazil) >60 years were cross-sectionally assessed through questionnaires, physical evaluations, laboratory tests, and full in-lab polysomnography (PSG). Three groups according to the OSA severity were compared according to sociodemographic characteristics, anthropometric measures, PSG parameters, the frequency of comorbidities, and the use of medications.

**Results:**

Participants' age ranged from 60 to 87 years with a mean of 70.02 ± 7.31, 59.8% female. In the univariate analysis, body mass index (BMI, kg/m^2^) (*p* = 0.049) and waist circumference (*p* = 0.005) were significantly higher in the participants with moderate OSA, but not among those with severe OSA. Participants with severe OSA had a higher arousal index (*p* = 0.007). Multivariate analysis showed that severe OSA was significantly associated with hypertension (*p* = 0.005), heart diseases (*p* = 0.025), and the use of two or more medications (*p* = 0.035).

**Conclusion:**

In a population-based study, severe, but not mild-to-moderate, OSA in older adults was associated with hypertension and the use of more medications. As age advances, anthropometric indicators of obesity may not increase the risk of severe OSA.

## Introduction

Aging process has been associated with marked changes in sleep parameters and an uprise in rates of sleep complaints in the older population (1, 2). Among the sleep disorders affecting aged individuals, obstructive sleep apnea (OSA) can reach frequencies the frequencies of 25–46% in population-based studies (2–5). The higher prevalence of OSA with aging may also be associated with non-related-to-weight factors, including morpho-functional changes in pharyngeal skeletal muscles (5–7). Although fat deposition has been related to OSA in general populations, indirect measurements have suggested lean mass reduction plays a significant role in the development of OSA among older adults (8). As a consequence, OSA leads to oxyhemoglobin desaturation, micro-arousals, and, eventually increased partial pressure of carbon dioxide in arterial blood (PaCO_2_) during sleep (9, 10), which are possible explanations for a higher risk of cognitive impairment, depression, metabolic disorders, cardiovascular disease, atrial fibrillation, and mortality (11). Additionally, clinical consequences can be different according to the classification of OSA severity defined by the apnea-hypopnea index (AHI, events/hour) as mild (AHI > 5 and <15), moderate (AHI > 15 and <30), or severe (AHI > 30 events). However, controversies remain whether current severity criteria is associated with higher risks of particularly in older adults (12, 13).

Although age *per se* has been considered a risk factor for OSA, evidence suggest OSA in older adults may be frequently less severe and OSA diagnostic criteria might be adjusted for this age group (7). Concurrently, it is likely the existence of a late-onset OSA presentation as a distinct phenotype has different pathophysiological mechanisms, as well as clinical manifestations and consequences, including mortality risk, according to its severity (14). In this study, we aimed to investigate clinical factors associated with OSA severity in older adults from a representative sample of the general population living in the city of São Paulo (15).

## Methods

### Study Characteristics

Data from the first follow-up of the São Paulo Epidemiologic Sleep Study (EPISONO), comprising a general population-based sample of São Paulo, Brazil, for this study. Baseline's survey design was comprehensively described elsewhere (16). In the first wave (from July to December 2007), 1,042 volunteers were included. From July 2015 to April 2016, the second wave of this study was carried out and included 715 individuals of the baseline sample. Of these participants in the second wave, we selected the older adults over 60 years of age, resulting in a sample of 199 participants. Follow-up participants were community-dwelling adults from both genders and derived from a multiethnic population. The Ethical Committee of the Universidade Federal de São Paulo approved this study (registration No. 0593/06), which was conducted according to the ethical standards defined in the 1964 Declaration of Helsinki as well as its subsequent amendments and was registered with ClinicalTrials.gov (identifier NCT00596713). Written informed consent was obtained from all volunteers.

### Sample Characterization

Sociodemographic and previous clinical characteristics were assessed *via* questionnaires. Previous clinical history of diseases and medications in use were checked when volunteers came to sleep lab. Participants were instructed to bring their medication lists or boxes to an in-person interview. Medications were checked and grouped according to their categories: cardiac medications, psychoactive medications, hypoglycemiants, and other.

All medical conditions and medications used by participants were checked through a personal interview. We considered the number of medications in use and classified according to the body system (nervous system, cardiovascular system, urinary system, metabolic, digester and nutritional system, bone and muscle system, etc.). Hypertension was considered according to a previous diagnosis; medication check or blood pressure higher than 140 × 90 mm Hg (17). Diabetes was also considered when a previous diagnosis and medication were taken or fasting glucose blood test was higher than 126 mg/dl (18).

Height (stadiometer to the nearest 0.1 cm) and weight (electronic scale to the nearest 0.1 kg) were used to calculate the body mass index (BMI, kg/m^2^). Circumference measures were part of the anthropometric evaluation, including neck circumference, abdominal circumference, and waist circumference.

### Sleep Measures and Classifications

Full in-lab polysomnography (PSG) was performed at the *Sleep Institute* (Department of Psychobiology/AFIP) using a digital EMBLA system (Medcare-Flaga hf. Medical Devices). The exam included electroencephalogram record, electrooculogram record, electromyogram of the submentonian muscles and tibial record, electrocardiogram (ECG) record, oronasal flow record, thoracic and abdominal movement, and record of snoring records, body position, and oximetry. Sleep efficiency was considered decreased when registered as below 85% and the rate of awakening was considered as normal when registered up to 10 events/h. An objective polysomnographic diagnosis of apnea was defined when the AHI was ≥5 obstructive events /hour and the occurrence of periodic leg movements (PLMs) more than 15 events/h (19). Participants were allocated into three groups: group 0 (G0), non- to mild OSA (*n* = 83); group 1 (G1), moderate OSA (*n* = 56); and group 2 (G2), severe OSA (*n* = 60).

### Statistical Analysis

Assumptions for statistical analysis were assessed by the Kolmogorov–Smirnov test, histograms, and Q–Q plots. The *Z*-score was used for the analysis of the outliers and was used for the variables with no normal distribution. The characteristics of participants were described using means and standard deviations (SDs) or absolute and relative frequency.

The *post-hoc* of Bonferroni was used. For multivariate regression analysis, the potential predictors for OSA were investigated by the Pearson correlation test and were excluded due to potential multicollinearity. Subsequently, linear regression modeling using enter method was performed. Having evaluated the relationships between the variables, multiple linear regression analysis was then performed to assess inflammatory profiles and comorbidities adjusted by anthropometric variables. For each output (dependent) variable, all the possible combinations of the predictor variables were assessed, with the lowest Akaike information criterion (AIC) used to select the strongest model. The Durbin–Watson (DW) test was used for an autocorrelation, and the variance inflation factor (VIF) and tolerance were performed to determine the presence of multicollinearity. We calculated tolerance and VIF values to evaluate multicollinearity between the variables, with tolerance >0.2 and VIF <10 considered indicative of no collinearity among the independent variables.

Contrasts were used to test for differences between the levels of a factor. The value of *p* < 0.05 was adopted as the criterion for statistical significance. All the analyses were performed using SPSS statistical software (version 24.0 for Windows).

## Result

Participant's age ranged from 60 to 87 years with a mean of 70.02 ± 7.31, allocated in three groups according to OSA severity. Arousal index was growing up according to the severity of OSA (*p* = 0.007). When stratified by gender, we noted a significant difference in N1 sleep. The percentage was higher in severe OSA. The N3 sleep was lower according to the severity of OSA in both genders (Table 1). No participants with central sleep apneas ≥5/h were found in out study.

**Table 1:** Sample characterization according to the presence and severity of OSA.

**Variable**		**Total**		**GO (non-OSA to mild OSA)**		**G1 (moderate OSA)**		**G2 (severe OSA)**		***P***
		***N***	**Mean ±SD**	***N***	**Mean ±SD**	***N***	**Mean ±SD**	***N***	**Mean ±SD**	
Age	Total	199	70.02 ± 7.31	83	69.76 ± 7.25	56	69.16 ± 6.75	60	71.17 ± 7.87	0.31
	Feminine	118	70.00 ± 7.50	56	69.55 ± 7.00	28	70, 10 ± 7.10	34	70.76 ± 7.8.70	0.76
	Male	81	70.00 ± 7.10	27	70.20 ± 7.7.90	28	68.25 ± 6.40	26	71.70 ± 7.6.70	0.20
BMI	Total	199	28.61 ± 5.39	83	28.86 ± 5.63	56	29.67 ± 5.80	60	27.26 ± 4.35	0.05
	Feminine	118	28.40 ± 5.50	56	29.45 ± 6.40	28	29.45 ± 6.40	34	26.90 ± 4.60	0.15
	Male	81	28.90 ± 5.22	27	29.00 ± 6.00	28	29.90 ± 5.30	26	27.72 ± 4.05	0.32
Neck C.	Total	199	36.10 ± 4.42	83	37.34 ± 3.99	56	38.85 ± 4.78	60	38.45 ± 4.56	0.09
	Feminine	118	38.214.36	56	37.57 ± 3.93	28	38.63 ± 4.75	34	28.93 ± 4.68	0.30
	Male	81	37.92 ± 4.54	27	36.86 ± 4.14	28	39.07 ± 4.88	26	37.80 ± 4.41	0.19
Waist C.	Total	199	99.67 ± 12.99	83	99.86 ± 13.26	56	103.58 ± 12.67	60	95.69 ± 11.91	0.004
	Feminine	118	100.20 ± 12.07	56	99.73 ± 11.36	28	103.11 ± 13.57	34	98.58 ± 11.84	0.31
	Male	81	98.88 ± 14.30	27	100.12 ± 16.77	28	104.04 ± 11.94	26	91.76 ± 11.05	0.005
Hip C.	Total	199	103.97 ± 10.74	83	104.20 ± 11.01	56	105.45 ± 11.07	60	102.22 ± 9.96	0.27
	Feminine	118	103.59 ± 9.38	56	104.04 ± 9.85	28	102.91 ± 82+98	34	103.4 ± 9.15	0.86
	Male	81	104.52 ± 12.52	27	104.53 ± 13.31	28	108.0 ± 12.47	26	100.63 ± 10.95	0.10
Efficiency (%)	Total	199	75.66 ± 32.18	83	79.95 ± 4, 642	56	75.38 ± 12.76	60	70.00 ± 1, 643	0.48
	Feminine	118	77.64 ± 39.46	56	73.18 ± 14.81	28	78.27 ± 9.11	34	84.73 ± 70.76	0.42
	Male	81	72.78 ± 16.50	27	72.48 ± 13.49	28	74.65 ± 16.07	26	71.09 ± 19.92	0.73
N1 (%)	Total	199	18.38 ± 12.36	83	11.25 ± 6.05	56	16.95 ± 8.51	60	27.54 ± 16.20	0.001
	Feminine	118	15.65 ± 9.46	56	12.25 ± 5.84	28	15.10 ± 9.27	34	21.69 ± 11.52	0.001
	Male	81	22.20 ± 14.84	27	13.73 ± 6.47	28	18.81 ± 7.37	26	35.20 ± 18.36	0.011
N2 (%)	Total	199	40.36 ± 9.44	83	42.66 ± 9.33	56	39.94 ± 7.57	60	37.56 ± 10.44	0.36
	Feminine	118	41.26 ± 8.85	56	39.34 ± 7.93	28	42.02 ± 8.33	34	43.79 ± 10.14	0.05
	Male	81	39.04 ± 10.15	27	38.74 ± 7.22	28	35.36 ± 10.59	26	43.33 ± 10.96	0.01
N3 (%)	Total	199	24.25 ± 10.80	83	25.98 ± 9.41	56	25.27 ± 9.89	60	20.89 ± 12.67	0.001
	Feminine	118	26.86 ± 10.08	56	26.60 ± 8.84	28	27.63 ± 9.77	34	26.65 ± 12.30	0.45
	Male	81	20.44 ± 10.74	27	24.71 ± 10.57	28	22.91 ± 9.60	26	22.75 ± 13.47	0.62
REM (%)	Total	199	17.00 ± 7.31	83	18.61 ± 6.45	56	17.82 ± 6.44	60	13.99 ± 8.32	0.007
	Feminine	118	16.59 ± 7.20	56	16.028 ± 7.66	28	15.66 ± 6.24	34	16.30 ± 7.05	0.25
	Male	81	17.58 ± 7.48	27	18.26 ± 6.70	28	17.33 ± 8.02	26	17.17 ± 7.87	0.85
Arousal Index	Total	199	28.55 ± 28.21	83	19.13 ± 8.77	56	24.1 ± 9.22	60	45.74 ± 45.17	0.007
	Feminine	118	26.80 ± 14.46	56	28.90 ± 14.62	28	26.94 ± 12.05	34	23.23 ± 15.68	0.19
	Male	81	27.02 ± 15.03	27	26.62 ± 13.34	28	33.43 ± 17.16	26	27.02 ± 15.03	0.07
Min SATO2 (%)	Total	199	83.00 ± 6.57	83	86.83 ± 4.49	56	82.39 ± 4.78	60	78.28 ± 7.20	0.14
	Feminine	118	83.27 ± 6.53	56	83.50 ± 6.66	28	81.03 ± 7.49	34	84.73 ± 4.97	0.07
	Male	81	82.61 ± 6.64	27	83.74 ± 6.59	28	81.92 ± 6.51	26	82.19 ± 6.93	0.56
AHI	Total	199	24.20 ± 19.51	83	7.71 ± 4.10	56	22.01 ± 4.42	60	49.53 ± 14.21	<0.001
	Feminine	118	22.35 ± 17.64	56	8.18 ± 4.07	28	21.83 ± 4.45	34	46.12 ± 11.67	0.00
	Male	81	27.24 ± 21.95	27	6.54 ± 4.0	28	22.06 ± 4.41	26	54.31 ± 15.83	0.00

*BMI, body mass index; Neck C, neck circumference; Waist C, waist circumference; Hip C, Hip circumference; SATO2, minimum oxygen saturation; AHI, apnea hypopnea index*.

*Date using mean and Standard deviation. P < 0.05 was considered significant*.

The use of medications was significantly higher according to OSA, with the highest severity among those classified as severe OSA (G2) (*p*: 0.035). Around 90% of older adults with severe OSA reported taking 2 or more medications, as shown in Table 2.

**Table 2:** Frequency of medication use by presence and severity of OSA.

			**G0** **(non-OSA to mild OSA)**	**G1** **(moderate OSA)**	**G2** **(severe OSA)**	**Total**	***P***
Medication Use	No	Total	23	12	6	41	0.035
		%	27.7%	21.4%	10.0%	20.6%	
	Yes	Total	60	44	54	158	
		%	72.3%	78.6%	90.0%	79.4%	

*P < 0.05 as considered significant*.

Table 3 depicts the multivariate analysis results. There was a significant association between neck circumference (*p*: 0.01), waist circumference (*p*: 0.031), hypertension (0.005), and heart problems (*p*: 0.025) with AHI. Of note, the inclusion of lipid metabolism, total cholesterol, LDL, HDL, and triglycerides did not change the final results.

**Table 3:** Associated factors with AHI.

**Model**	**Standardized coefficient Beta**	**t**	**Sig**.
(Constante)		−0.042	0.967
BMI	−0.023	−0.206	0.837
Epworth	0.069	0.963	0.337
Neck Circumference	0.325	3.520	0.001
Waist Circumference	−0.293	−2.168	0.031
Glycaemia	−0.071	−0.979	0.329
Hypertension	0.203	2.863	0.005
Diabetes	−0.011	−0.153	0.878
Heart disease	0.157	2.265	0.025
Hypothyroidism	0.111	1.578	0.116
Kidney disease	−0.035	−0.497	0.620
Osteoporosis	−0.066	−0.922	0.358
Depression	−0.050	−0.712	0.478

*Dependent variable: AHI, Linear regression: R^2^ = 0.21*.

## Discussion

This study evaluated whether the frequently described associations between OSA and clinical comorbidities were present in the sample of older adults from a population-based cohort. Among our findings, severe OSA was not associated with anthropometric indicators of obesity, including BMI and waist circumference. On the other hand, those with severe OSA were more likely to have hypertension and to use two or more medications, as well as to have a higher arousal index. Severe OSA in this aged sample was not associated with other commonly described OSA-related comorbidities, such as diabetes. However, when performing regression analysis considering AHI as a whole, neck and waist circumferences, and waist were significantly with increased AHI.

Obesity is characterized as the main risk factor for OSA and is present in approximately 60% of apneic individuals (20–22). According to the literature, the distribution of body fat plays an important role in this disorder, with visceral obesity being the most harmful, followed by an increase in neck circumference, considered a robust predictor (23) as also seen in our study. Although the BMI is considered as a risk factor for severe OSA in young and middle-aged adults, we have not found this association in from our study. The exact mechanisms whereby aging increases the risk of severe OSA are not completely understood in aged individuals (24, 25). Anatomically small pharyngeal and a compromised pharyngeal anatomy are the key factors in the development of an upper airway obstruction during sleep (26, 27). In addition to the anatomic characteristics, pulmonary and muscle-related mechanisms also play important role in the mechanism of OSA, and it has already been shown that these mechanisms are changed in older population (28, 29), which could justify higher frequencies of OSA independently of the obesity status in this age group.

Airway anatomy/collapsibility thus may play a pathogenic role in older adults, whereas a sensitive ventilator control system is a more prominent trait in younger adults with OSA (30).

With regard to cardiovascular consequences of OSA in the elderly, there has been an also controversial finding.

Aging by itself is a well-known risk factor for hypertension (31). Some studies have shown that the risk of hypertension and CVD related to OSA diminishes with aging (32). Our study conducted in the general population showed that severe OSA was associated with hypertension particularly in the older population. OSA and arterial hypertension are often concomitant conditions and are associated with an increased cardiovascular risk (33, 34).

A recent meta-analysis, involving 20 original studies demonstrated that OSA increases the risk of hypertension in a dose-response manner (35). The pathogenesis of hypertension in OSA is complex and not fully understood. Potential pathophysiological mechanisms increasing the risk of hypertension among people with OSA include intermittent hypoxia with endothelial dysfunction, sleep fragmentation, nocturnal fluid shift, and the activation of renin–angiotensin–aldosterone (RAA) system. OSA may play a role in the sympathetic activation and increased oxidative stress associated with the hypertension pathogenesis (36, 37). Our findings showing an increase in hypertension frequency among older adults with OSA suggest that similar mechanisms might also be present in advanced age groups.

Medication use and polypharmacy vary according to the demographic characteristics and tend to increase with age and the presence of comorbidities. The average number of medications used by the older adults ranges from two to five medicines (37, 38). The polypharmacy is common in the elderly, and many medications may contribute to sleep disturbances. In addition to medicines oftentimes, Additionally, older adults have various comorbid conditions that interfere with sleep, such as depression, prostate hypertrophy, gastroesophageal reflux, arthritis, and pulmonary conditions (39).

Although the literature brings other comorbidities, such as diabetes and depression as associated with the severity of sleep apnea in adults, we did not find this association in our sample (40, 41). The role of OSA as an independent risk factor for comorbidities remains controversial due to the presence of powerful confounders, such as aging, gender, lifestyle factors and comorbidities (42, 43).

A German population-based study investigated the prevalence of OSA in different age and gender groups. The continuous increase of OSA with age challenges the current theory that mortality due to OSA and cardiovascular comorbidities affects the prevalence of OSA at an advanced age (44). The pathophysiology of OSA possible dysfunction of the air muscles, resulting from craniofacial abnormalities or higher age-related upper airway collapse. It is speculated that the hereditability related to the development of OSA is close to 40% whilst environmental and lifestyle factors account for 60% of the pathophysiological factors (45).

Obesity is undoubtedly one of the most significant risk factors as it increases the risk by 10–14 times for OSA and weight loss reduces the risk of obesity for this condition (46). However, we did not find obesity as a significant risk among severe OSA in older individuals of our population-based study, which is consistent with the findings of other authors and can be justified by differences in other than obesity-related risk factors for OSA among those >60 years (including hormonal and inflammatory changes, comorbidities, and other diseases) (47, 48).

Studies have reported that the increase in mortality in patients with OSA from cardiovascular causes was only seen in patients under the age of 50 and when the sleep-disordered breathing was severe (49), whereas in elderly with sleep apnea the mortality rates by cardiovascular disease were similar to those of the elderly without sleep apnea (50, 51). Interestingly, there is evidence that OSA in older populations has a better prognosis, which may be explained by the marked cardiovascular response in middle-aged patients, which is not observed in older populations (52).

To explain these discrepancies between mortality and sleep apnea in the elderly, Kobayashi et al. proposed that there are two clinically different phenotypes of OSA in the older population: one in which the disease appears in middle age and has more symptoms and consequences; and another in which it appeared in old age and is less serious (53). Thus, the authors suggest that cardiovascular risks are not uniformly associated with OSA among older adults.

However, a major potential confounder in the association between OSA and mortality is the co-occurrence of comorbidities. Several studies have shown that cardiovascular morbidity and mortality are significantly higher in severe OSA and accompanied by other comorbidities (54, 55). Whether a possible bidirectional influence of medical comorbidities and OSA is a subject of better designed studies.

Lavie and Lavie hypothesized that an apparent decline in mortality risk with age could be explained by an adaptive protective mechanism resulting from nightly cycles of hypoxia–reoxygenation with ischemic preconditioning (56). Ischemic preconditioning is related to gene expression programs (HIF-1α), and *in vitro* study in mice subjected to intermittent hypoxia was protected against post-ischemic damage, mediated by HIF-1α (57).

Similar to sleep apnea, vascular endothelial growth factor (VEGF) production increases in a gravity-dependent manner and appears to protect the myocardium during periods of severe ischemia with myocardial neovascularization (58, 59). Hypothetically, some patients will be protected against major ischemic events by producing sufficiently high concentrations of VEGF to induce the formation of collateral vessels that guarantee sufficient blood supply to the myocardium in case of significant obstruction of the coronary arteries. In summary, apneic events promote long-term cardiovascular protection by activating the genetic programs that induce vascular remodeling and other protective responses (56). However, considering the similar pathophysiological mechanisms for the development of hypertension, this theory is not entirely consistent with the higher hypertension risk found in our study.

This study has some limitations. The cross-sectional design does not allow causal inferences. The assessment of medical conditions relied on self-report although all information was reconfirmed as well as all the medicines used throughout an in-person interview. The standardization of hypopneas definition has changed over time, and so has the recent definition adopted by the American Academy of Sleep Medicine. This definition can influence to a greater or lesser degree of the main discretion of OSA severity, which is given by the AHI per hour of sleep (19). The lack of standardization on the discretion of OSA severity in the elderly is still under debate.

## Implications

Studies have shown that an estimated 80–90% of people with OSA remain undiagnosed and untreated (60).

Obstructive sleep apnea in the elderly seems not to be a benign condition as it has also been associated with higher healthcare utilization. Systematic surveys, however, that characterize the continuum of disordered breathing during sleep and the associated health impairment in the older population are not yet available (61, 62).

In our study, the association of OSA with hypertension, medication, and a higher arousal index suggests a possible bidirectional association between OSA and comorbidities. These findings emphasize the importance of thorough phenotype evaluation regarding possible gender and age-specific differences in OSA pathogenesis, mechanism, and susceptibility. In a next step, we recommend further investigation of the influence of age and all alterations on the prevalence of OSA. It is becoming more and more important to investigate not only OSA defined purely by the AHI but also OSA syndrome and its symptoms as an apparent disease (63). Therefore, it is highly relevant (especially considering the high prevalence) to improve definitions and therapy strategies.

## Data Availability Statement

The original contributions presented in the study are included in the article/supplementary material, further inquiries can be directed to the corresponding author.

## Ethics Statement

The studies involving human participants were reviewed and approved by Ethical Committee of the Federal University of São Paulo. The patients/participants provided their written informed consent to participate in this study.

## Author Contributions

All authors listed have made a substantial, direct, and intellectual contribution to the work and approved it for publication.

## Funding

This work was funded by AFIP (Associação Fundo de Incentivo à Pesquisa) for funding the study and CAPES (Coordenação de Aperfeiçoamento de Pessoal de Nível Superior) PRINT grants number (88887.194874/2018-00).

## Conflict of Interest

The authors declare that the research was conducted in the absence of any commercial or financial relationships that could be construed as a potential conflict of interest.

## Publisher's Note

All claims expressed in this article are solely those of the authors and do not necessarily represent those of their affiliated organizations, or those of the publisher, the editors and the reviewers. Any product that may be evaluated in this article, or claim that may be made by its manufacturer, is not guaranteed or endorsed by the publisher.
